# Supplementary material for: APOBEC3B reporter myeloma cell lines identify DNA damage response pathways leading to APOBEC3B expression
Source: PLoS One. 2020 Jan 8;15(1):e0223463. doi: 10.1371/journal.pone.0223463 (PMC6948746; doi:10.1371/journal.pone.0223463)

Supplementary information: Original membranes and gels

Original gel for Fig 2A

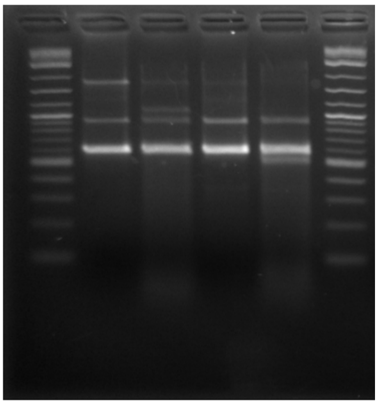

Original gel for Figs 2C and 2D

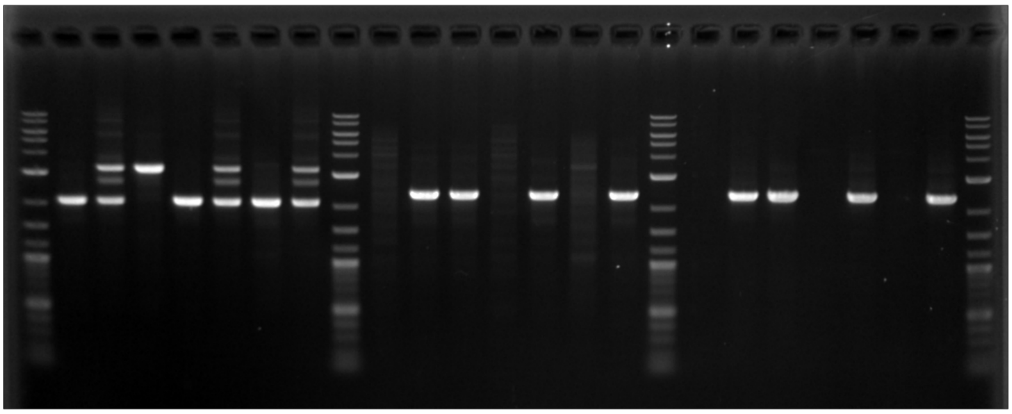

Original membranes for Fig 2G

anti-FLAG

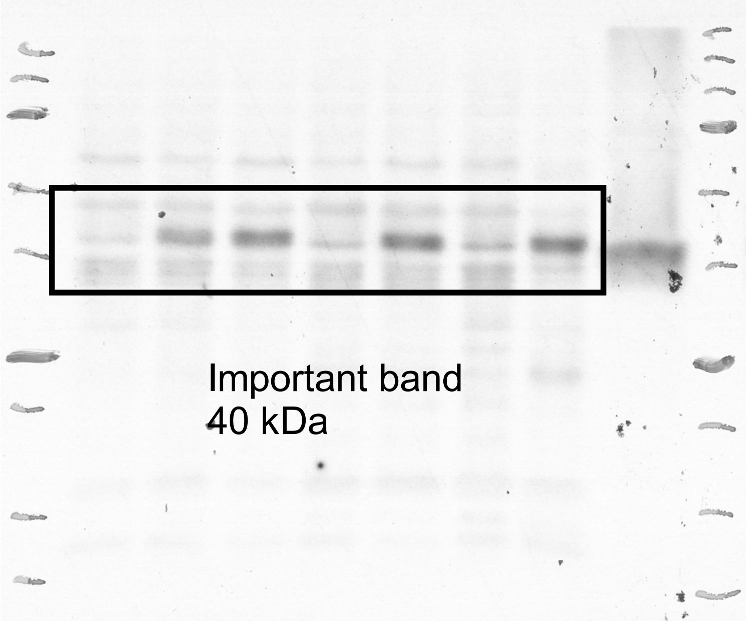

anti-FLAG

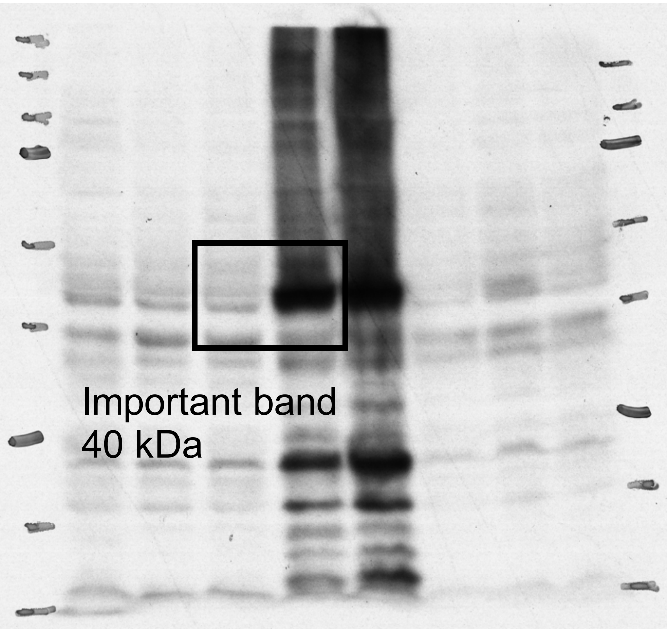

anti-α-Tubulin

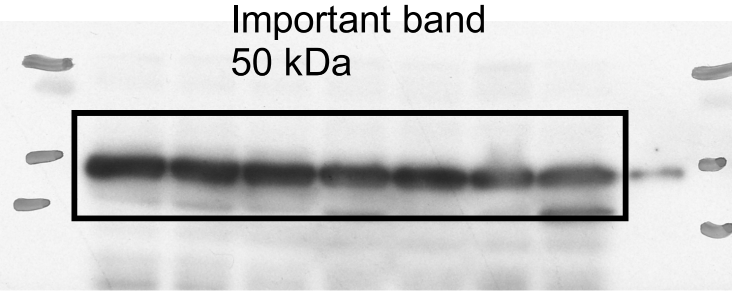

anti-α-Tubulin

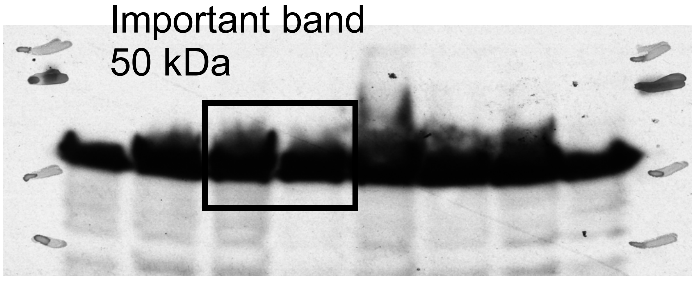

Original membranes for Fig 3B

anti-FLAG

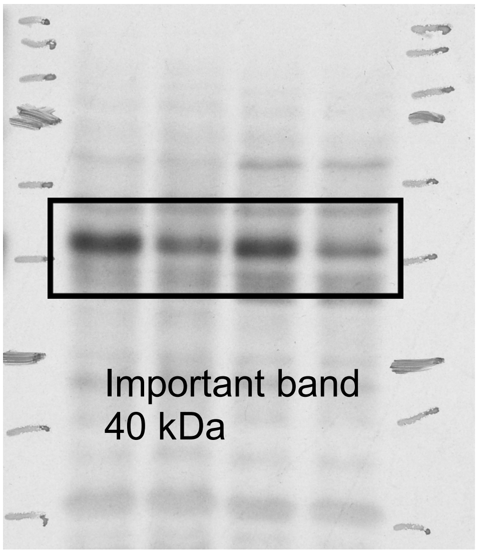

anti-FLAG

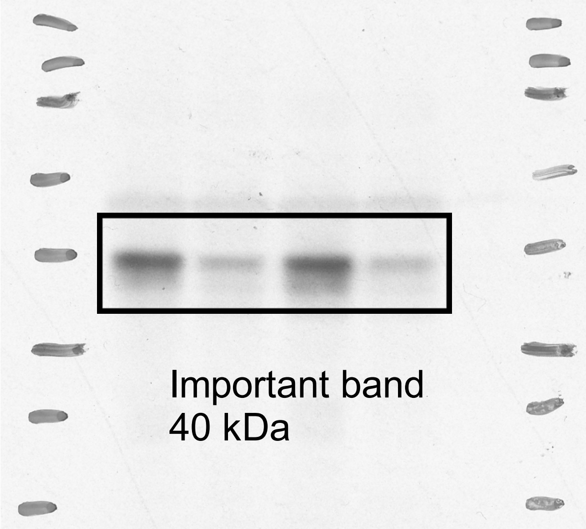

anti-α-Tubulin

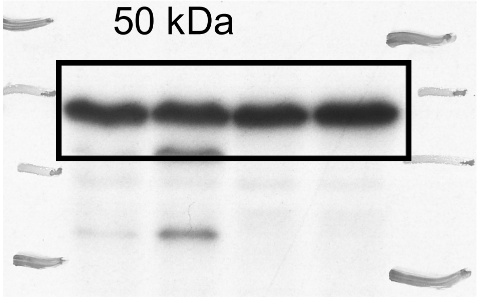

anti-α-Tubulin

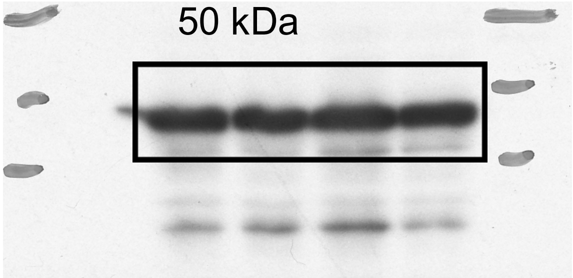

Original membranes for Fig 3H

anti-FLAG

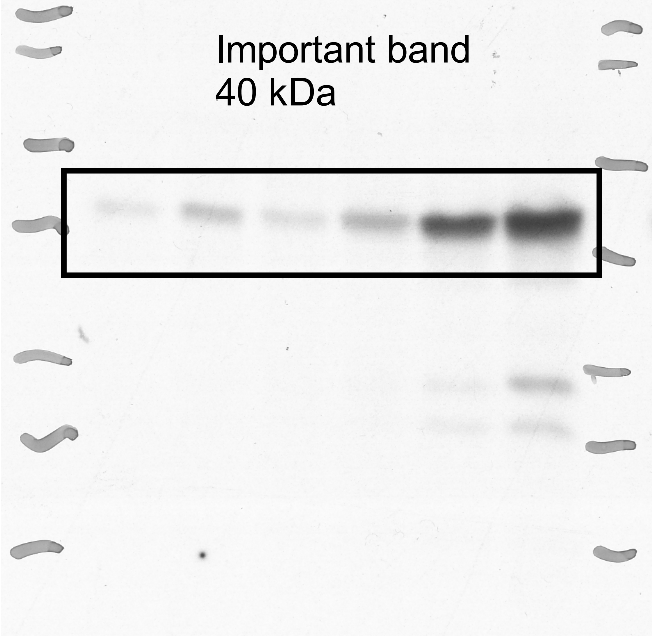

anti-α-Tubulin

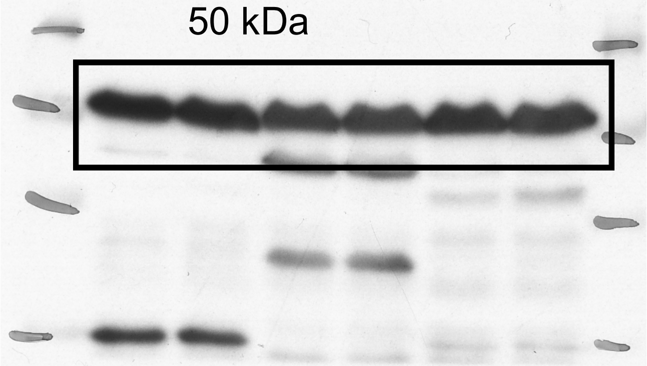

Supplement: S1 Fig — (PDF) [file pone.0223463.s001.pdf]
